# Association between neck circumference and glucose tolerance levels at 2-6 months postpartum in women with and without gestational diabetes

**DOI:** 10.20945/2359-4292-2022-0242

**Published:** 2024-06-03

**Authors:** Camila Rodrigues de Souza Carvalho, Patricia Medici Dualib, Juliana Ogassavara, Rosiane Mattar, Sérgio Atala Dib, Bianca de Almeida-Pititto

**Affiliations:** 1 Universidade Federal de São Paulo São Paulo SP Brasil Programa de Pós-graduação em Endocrinologia e Metabologia, Universidade Federal de São Paulo ão Paulo, SP, Brasil; 2 Universidade Federal de São Paulo Departamento de Medicina São Paulo SP Brasil Departamento de Medicina, Universidade Federal de São Paulo, São Paulo, SP, Brasil; 3 Universidade Federal de São Paulo Departamento de Obstetrícia São Paulo SP Brasil Departamento de Obstetrícia, Universidade Federal de São Paulo, São Paulo, SP, Brasil; 4 Universidade Federal de São Paulo Departamento de Medicina Preventiva São Paulo SP Brasil Departamento de Medicina Preventiva, Universidade Federal de São Paulo São Paulo, SP, Brasil

**Keywords:** Neck circumference, pregnancy, glucose intolerance, type 2 diabetes mellitus, gestational diabetes

## Abstract

**Objective:**

To evaluate the association between neck circumference (NC) measured during pregnancy and markers of glucose metabolism measured 2-6 months postpartum in women with overweight/obesity with and without gestational diabetes (GDM).

**Subjects and methods:**

This prospective study enrolled 100 pregnant women (including 50 with GDM) with pregestational body mass index (BMI) ≥ 25 kg and < 40 kg/m². The cohort was stratified according to NC tertiles during pregnancy. Glucose metabolism was assessed in the postpartum period. The association between NC during pregnancy and markers of glucose metabolism postpartum was tested using linear regression analysis.

**Results:**

Participants with NC in the third tertile, compared with those with NC in the second and first tertiles, had higher levels of glycated hemoglobin (HbA1c; 5.6 ± 0.4% *versus* 5.4 ± 0.3% *versus* 5.3 ± 0.2%, respectively, p = 0.006), fasting insulin (13.2 ± 6.6 µIU/mL *versus* 11.1 ± 5.8 µIU/mL *versus* 9.5 ± 4.9 µIU/mL, respectively, p = 0.035), homeostasis model for insulin resistance (HOMA-IR; 3.1 ± 1.7 *versus* 2.5 ± 1.3 *versus* 2.1 ± 1.2, respectively, p = 0.035) and triglyceride-glucose index (TyG; 4.6 ± 0.2 *versus* 4.5 ± 0.2 *versus* 4.5 ± 0.3, respectively, p = 0.010). In crude linear regression analysis, NC measured during pregnancy was significantly associated with levels of fasting plasma glucose, 2-hour glucose, HbA1c, log HOMA-IR, and TyG index. The association remained after adjustment for age, family history of diabetes, and number of pregnancies. When adjusted for pregestational BMI and gestational weight gain, NC remained independently associated with fasting plasma glucose and HbA1c levels.

**Conclusion:**

The NC measured during pregnancy was positively associated with worse glucose metabolic profile in the postpartum among women with obesity/overweight with and without GDM. The NC measurement may be a feasible tool for early identification of women at a higher risk of developing type 2 diabetes mellitus.

## INTRODUCTION

Type 2 diabetes mellitus (T2DM) represents an important cause of morbidity and mortality globally, currently affecting 536 million adults and projected to increase to 783 million in 2045 (1,2). Given this profound impact, it is critical to identify risk factors associated with the development of T2DM, with a focus on preventive measures and effective disease control.

An important risk factor for the development of T2DM is hyperglycemia during pregnancy and, particularly, gestational diabetes (GDM), as pregnant women with GDM are more likely to develop T2DM (3). Additionally, GDM is associated with concerning epidemiological data and is one of the most common complications in the gestational period. Notably, about 18% of pregnant women in Brazil have hyperglycemia (4).

Excessive weight is also an important risk factor for both T2DM and GDM, considering its central role in the pathophysiology of insulin resistance and high prevalence in the population and among women in fertile age (4,5). In this context, most women with GDM are overweight or obese (6), and it is difficult to identify those who could progress to having altered glucose metabolism after delivery. Better screening of pregnant women at greater risk for T2DM could help improve prevention strategies. Traditional insulin resistance markers, which are components of the metabolic syndrome (*e.g.*, increased measures of central adiposity such as waist circumference, high triglyceride levels, low high-density lipoprotein [HDL] cholesterol levels, and hypertension), can identify T2DM risk, but are difficult to evaluate during pregnancy. Indeed, the measurement of waist circumference is impracticable during pregnancy. Also, body mass index (BMI) is not a good assessment of body fat distribution or a reliable parameter in pregnancy, when weight increases progressively.

The neck circumference (NC), an anthropometric measurement, has gained attention due to its ease of measurement and association with cardiometabolic risk factors such as insulin resistance, central obesity, blood pressure, postprandial glucose levels, and triglycerides (7,8). Measurement of NC may be interesting during pregnancy, as other markers adopted for the general population and for women in the fertile period lose their effectiveness (*e.g.*, waist circumference and BMI for assessment of overweight or obesity) (9,10). Notably, studies have shown an association of NC with GDM during pregnancy but not with altered plasma glucose levels in the postpartum period (8-12).

Considering these scenarios, the present study aimed to evaluate the association of NC measured during the first and third trimesters of pregnancy with markers of glucose metabolism (levels of fasting plasma glucose, fasting insulin, glycated hemoglobin [HbA1c], and indices of insulin resistance) at 2-6 months in the postpartum period in women with overweight/obesity with and without GDM.

## SUBJECTS AND METHODS

### Study population and design

From September 2018 to December 2019, all pregnant women with a BMI > 25 kg/m^2^ who attended the general gestation outpatient clinic of the Obstetrics Division and the gestational diabetes outpatient clinic of the Diabetes Center at Federal University of São Paulo (SP, Brazil) were invited to participate in the present study. The ethics committee of the Federal University of São Paulo approved the study, and all participants signed a consent form (Certificate of Presentation for Ethical Appreciation [Unifesp] CAAE 06745219.8.0000.5505).

The eligibility criteria were age ≥ 18 years, BMI ≥ 25 kg/m² but < 40 kg/m² at any pregnancy trimester, and absence of known autoimmune diseases or chronic use of medications. A total of 143 pregnant women were included in the study, and 132 completed the evaluation during pregnancy (including 61 with GDM). For the diagnosis of GDM, we use the criteria from the International Association of Diabetes and Pregnancy Study Groups (IAPDSG), which are similar to those of the Brazilian GDM guidelines (13). In the postpartum period (60-180 days after delivery), 100 women attended the study evaluation (including 50 with GDM).

**Figure 1:**
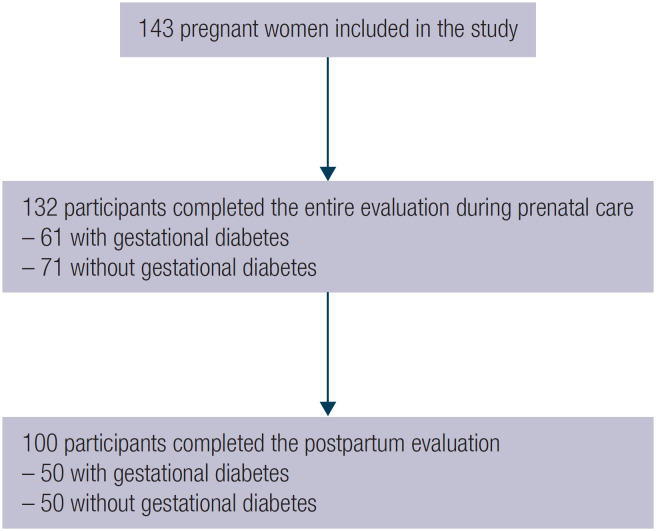
Flow diagram of the participants' inclusion in the study

The study had a longitudinal design and included the assessment of the participants during pregnancy and in the postpartum period (Figure 1). At each pregnancy trimester and in the postpartum, the participants were evaluated with standardized questionnaires and their anthropometric data and laboratory tests were collected.

#### Standardized questionnaires

Using standardized questionnaires, postpartum information was obtained under supervision of trained interviewers. The data collected included the pregestational BMI, amount of weight gained during pregnancy, number of previous pregnancies, gestational week at delivery, method of delivery, occurrence of maternal and fetal complications, and use of medications. A high education level was defined by at least 14 years of schooling. Physical activity levels during pregnancy and in the postpartum period were deemed to be of moderate intensity when lasting ≥ 75 minutes/week and high intensity when lasting ≥ 150 minutes/week.

#### Anthropometry and blood pressure

Weight was measured using a digital scale (Rice Lake, São Paulo, Brazil) accurate to 100 g. Height was measured using a portable stadiometer accurate to 0.5 cm and equipped with a sliding headboard positioned against a wall at a 90° angle to the floor. The participants were instructed to remove shoes and socks, following which the observer positioned the sliding headboard to align with the participant's vertex. Both these measurements were used to calculate each participant's BMI. The participants’ NC (in cm) was measured using an inelastic tape immediately below the cricoid cartilage and perpendicular to the neck's long axis, with the participant seated. The waist circumference (in cm) was measured using a flexible tape between the iliac crest and the last ribs. Using a mercury sphygmomanometer adjusted to the brachial circumference, blood pressure was obtained three times after a 5-minute rest with the patient in the sitting position. The final systolic and diastolic pressure values was the average of the last two measurements.

The cohort was stratified according to tertiles of NC during pregnancy, specifically, first tertile (NC ≤ 33.3 cm), second tertile (NC 33.4-36.3 cm), and third tertile (NC ≥ 36.4 cm). The average NC measured in the first trimester and third trimester were compared, and as it showed no significant difference (p = 0.43) (14), we opted to use the analysis of the NC evaluated in the second/third trimester.

#### Laboratory tests

All pregnant women were invited to undergo laboratory and clinical evaluation in the postpartum period (60-180 days after delivery) according to a predefined schedule. Plasma samples were collected after overnight fasting, and a 75-g oral glucose tolerance test (OGTT) was performed with measurement of plasma glucose at 2 hours. The samples were immediately centrifuged and analyzed by a private and certificate laboratory. Plasma glucose was determined using the glucose oxidase method. The concentrations of total cholesterol, HDL cholesterol, and triglycerides were determined through enzymatic colorimetric assays using an automated analyzer. Levels of low-density lipoprotein (LDL) cholesterol and very-low-density lipoprotein (VLDL) cholesterol were estimated using the Friedewald equation. Insulin was measured using the chemiluminescence method.

Insulin resistance was evaluated using the homeostatic model of insulin resistance (HOMA-IR) and the triglyceride-glucose index (TyG) according to the following equations:

HOMA-IR = (fasting insulin in µIU/mL x fasting glucose in mmol/L) / 22.5

TyG index = log (fasting triglycerides in mg/dL x fasting glucose in mg/dL) / 2

The diagnostic criteria of T2DM were fasting plasma glucose ≥ 126 mg/dL, plasma glucose 2 hours after 75 g of glucose ≥ 200 mg/dL, or HbA1c ≥ 6.5% (13).

#### Statistical analysis

Continuous variables were presented as mean ± standard deviation and categorical variables as frequency (%). Clinical and laboratory variables were compared using analysis of variance (ANOVA) for continuous variables or the chi-square test for categorical variables according to NC tertiles. The association between NC during pregnancy (independent variable of main interest) with continuous variables of glucose metabolism and insulin resistance in the postpartum (dependent variables) was tested using linear regression analysis with a crude model and with models adjusted for age, family history of diabetes, number of pregnancies (Model 1), pregestational BMI and weight gain during pregnancy (Model 2), and GDM (Model 3). A comparison of relevant characteristics between women who attended the postpartum evaluation and those who were lost to follow-up was also performed and is presented in the Supplementary Table. All statistical analyses were performed using the software Statistical Package for the Social Sciences, Version 22.0 (IBM Corp. IBM Corp., Armonk, NY, USA). P levels < 5% were considered statistically significant.

## RESULTS

The sample included 100 participants evaluated during the postpartum period, stratified according to tertiles of NC during pregnancy, specifically, first tertile (n = 33; NC ≤ 33.3 cm), second tertile (n = 34; NC 33.4-36.3 cm), and third tertile (n = 33; NC ≥ 36.4 cm).

Table 1 shows the participants’ characteristics obtained before, during, and after pregnancy, categorized according to NC tertiles. A comparison of pregestational data showed that all three NC tertile groups had comparable age, ethnicity, education level, family history of diabetes, and physical activity level. Women in the third tertile (*i.e.*, those with NC ≥ 36.4 cm), compared with those in the second and first tertiles, had higher pregestational weight (85.9 ± 10.9 kg *versus* 74.0 ± 9.6 kg *versus* 70.7 ± 8.6 kg, respectively, p < 0.001) and BMI (32.7 ± 3.8 kg/m² *versus* 28.6 ± 3.0 kg/m² *versus* 27.6 ± 2.8 kg/m², respectively, p < 0.001).

**Table 1:** Participants’ characteristics obtained before, during, and after pregnancy, categorized according to tertiles of neck circumference

	First tertile (NC ≤ 33.3 cm)	Second tertile (NC 33.4-36.3 cm)	Third tertile (NC ≥ 36.4 cm)	P values
**Pregestational data**				
Age (years)	29.0 ± 6.8	31.9 ± 6.6	31.0 ± 6.1	0.206
White ethnicity – n (%)	15 (45.5)	11 (32.4)	18 (54.5)	0.184
High education level – n (%)	11 (33.3)	6 (17.6)	9 (37.3)	0.336
Family history of DM – n (%)	8 (25.0)	16 (47.1)	10 (30.3)	0.141
Physically active – n (%)	14 (42.4)	14 (41.2)	14 (42.4)	0.993
Weight (kg)	70.7 ± 8.6	74.0 ± 9.6	85.9 ± 10.9^a^^b^	<0.001
BMI (kg/m²)	27.6 ± 2.8	28.6 ± 3.0^a^	32.7 ± 3.8^a^^b^	<0.001
Number of pregnancies – n (%)	8 (24.2)	14 (41.2)	15 (45.5)	0.168
**Gestational data**				
**First and second trimesters**				
Weight (kg)	73.0 ± 9.8	78.2 ± 9.8	91.5 ± 10.3^a,b^	<0.001
Systolic blood pressure (mmHg)	108.7 ± 12.6	111.5 ± 11.3	114.7 ± 11.3	0.194
Diastolic blood pressure (mmHg)	67.4 ± 10.2	67.8 ± 9.6	71.4 ± 10.3	0.307
**Third trimester**				
Weight (kg)	77.7 ± 8.4	82.0 ± 10.0	92.0 ± 11.5^a^^b^	<0.001
Systolic blood pressure (mmHg)	108.4 ± 11.1	113.0 ± 10.3	119.8 ± 11.7^a^	<0.001
Diastolic blood pressure (mmHg)	66.2 ± 8.3	71.9 ± 9.5	74.9 ± 11.0^a^	0.002
Gestational weight gain (kg)	9.6 ± 6.1	10.5 ± 5.6	9.5 ± 6.3	0.774
Gestational age of delivery (weeks)	38.6 ± 1.6	38.8 ± 1.1	38.4 ± 1.1	0.508
Birth weight (kg)	3.2 ± 0.6	3.2 ± 0.4	3.3 ± 0.4	0.640
Birth weight according to gestational age – n (%)				
Small for gestational age	3 (9.1)	1 (3)	3 (0.4)	0.306
Adequate for gestational age	25 (75.8)	28 (84.8)	20 (62.5)	
Large for gestational age	5 (12.2)	4 (12.1)	9 (28.1)	
GDM – n (%)	9 (27.3)	18 (52.9)	23 (69.7)	0.002
Insulin use during pregnancy – n (%)	4 (12.1)	9 (27.3)	16 (50)	0.003
**Postpartum data**				
Physically active – n (%)	0 (0.0)	2 (5.9)	6 (6.3)	0.352
Weight (kg)	71.4 ± 9.3	75.3 ± 11.6	85.6 ± 11.5^a,b^	<0.001
BMI (kg/m²)	27.4 ± 3.1	29.4 ± 3.7	32.1 ± 3.6^a,b^	<0.001
Neck circumference (cm)	33.16 ± 1.32	34.8 ± 2.0^a^	37.4 ± 2.6^a,b^	<0.001
Systolic blood pressure (mmHg)	110.6 ± 9.6	117.2 ± 8.7^a^	115.50 ± 9.50	0.013
Diastolic blood pressure (mmHg)	70.7 ± 6.9	75.4 ± 7.5^a^	74.5 ± 5.9	0.016
Total cholesterol (mg/dL)	194.7 ± 38.2	190.5 ± 49.5	194.4 ± 40.0	0.904
LDL cholesterol (mg/dL)	116.6 ± 34.3	113.1 ± 43.0	117.4 ± 35.2	0.887
HDL cholesterol (mg/dL)	56.5 ± 12.0	56.2 ± 9.1	52.2 ± 12.0	0.230
Triglycerides (mg/dL)	109.4 ± 68.6	93.5 ± 47.8	124.0 ± 43.1^b^	0.018
Fasting plasma glucose (mg/dL)	90.6 ± 8.6	90.2 ± 10.2	95.3 ± 14.1	0.131
2-hour plasma glucose (mg/dL)	105.8 ± 34.8	99.6 ± 19.6	119.8 ± 42.0	0.050
HbA1c (%)	5.3 ± 0.2	5.4 ± 0.3	5.6 ± 0.4^a, b^	0.006
Fasting insulin (µIU/mL)	9.5 ± 4.9	11.1 ± 5.8	13.2 ± 6.6^a^	0.041
2-hour insulin (µIU/mL)	42.4 ± 39.7	44.4 ± 29.0	57.2 ± 39.6	0.230
HOMA-IR	2.1 ± 1.2	2.5 ± 1.3	3.1 ± 1.7^a^	0.035
TyG index	4.5 ± 0.3	4.5 ± 0.2	4.6 ± 0.2^b^	0.010
Prediabetes – n (%)	7 (23.3)	7 (21.2)	9 (28.1)	0.802

The values are shown as mean ± standard deviations or n (%). Analysis of variance (ANOVA) was used for continuous variables, and the chi-square test was used for categorical variables. Variables without normal distribution were log transformed. Bonferroni correction was applied if p < 0.05.

a ,*versus* first tertile.

b ,*versus* second tertile. A high education level was defined by at least 14 years of schooling. Physical activity levels were deemed to be of moderate intensity when lasting ≥ 75 minutes/week and high intensity when lasting ≥ 150 minutes/week. Abbreviations: BMI, body mass index; DM, diabetes mellitus; GDM, gestational diabetes; HbA1c, glycated hemoglobin; HDL, high-density lipoprotein; HOMA-IR, homeostatic model of insulin resistance; LDL, low-density lipoprotein; NC, neck circumference; TyG index, triglyceride-glucose index. The HOMA-IR index was calculated using the formula (fasting insulin in µIU/mL x fasting glucose in mmol/L) / 22.5 and the TyG index was calculated as log (fasting triglycerides in mg/dL X fasting glucose in mg/dL) / 2.

During pregnancy, women in the third tertile of NC, compared with those in the second and first tertiles, had greater mean weight during the first and second trimesters (91.5 ± 10.3 kg *versus* 78.2 ± 9.8 kg *versus* 73.0 ± 9.8 kg, respectively, p < 0.001) and third trimester (92.0 ± 11.5 kg *versus* 82.0 ± 10.0 kg *versus* 77.7 ± 8.4 kg, respectively, p < 0.001). Differences between the third, second, and first tertiles were also observed in terms of mean systolic blood pressure levels (119.8 ± 11.7 mmHg *versus* 113.0 ± 10.3 mmHg *versus* 108.4 ± 11.1 mmHg, respectively, p < 0.001) and mean diastolic blood pressure levels (74.9 ± 11.0 mmHg *versus* 71.9 ± 9.5 mmHg *versus* 66.2 ± 8.3 mmHg, respectively, p = 0.002).

A comparison between the third, second, and first tertiles in the postpartum period showed persistent differences between groups regarding mean weight (85.6 ± 11.5 kg *versus* 75.3 ± 11.6 kg *versus* 71.4 ± 9.3 kg, respectively, p < 0.001) and mean BMI (32.1 ± 3.6 kg/m² *versus* 29.4 ± 3.7 kg/m² *versus* 27.4 ± 3.1 kg/m², respectively, p < 0.001). In the postpartum period, the mean NC increased progressively across tertiles, from 33.16 ± 1.32 cm in the first tertile to 34.8 ± 2.0 cm in the second tertile and 37.4 ± 2.6 cm in the third tertile (p < 0.001). Additionally, the second tertile group, compared with the third and first tertile groups, had higher mean levels of systolic (117.2 ± 8.7 mmHg *versus* 115.50 ± 9.50 mmHg *versus* 110.6 ± 9.6 mmHg, respectively, p = 0.013) and diastolic (75.4 ± 7.5 mmHg *versus* 74.5 ± 5.9 mmHg *versus* 70.7 ± 6.9 mmHg, respectively, p = 0.016) blood pressure. Finally, the third tertile group, compared with the second and first tertile groups, had higher levels of triglycerides (124.0 ± 43.1 mg/dL *versus* 93.5 ± 47.8 mg/dL *versus* 109.4 ± 68.6 mg/dL, respectively, p = 0.018).

**Figure 2:**
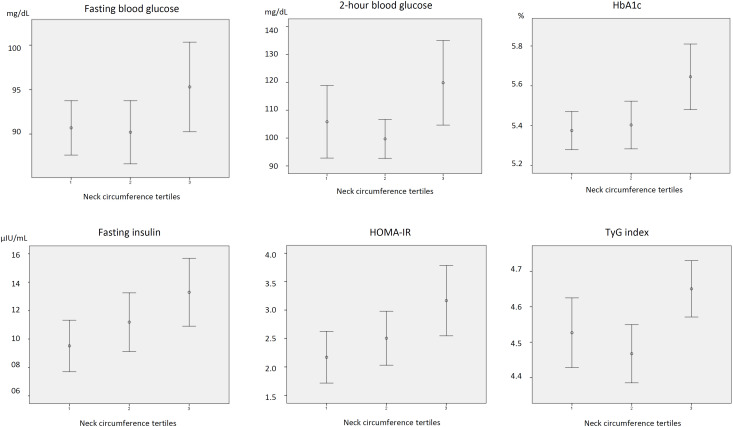
Mean levels and 95% confidence intervals of fasting and 2-hour glucose, fasting insulin, glycated hemoglobin, homeostatic model of insulin resistance, and triglyceride-glucose index according to tertiles of neck circumference at 2 to 4 months after delivery in women with overweight during pregnancy or gestational diabetes.

Regarding plasma glucose levels in the postpartum period, the mean 2-hour glucose did not differ between groups but had a borderline statistical significance (p = 0.05). Additionally, the third tertile group, compared with the second and first tertile groups, had higher mean levels of HbA1c (5.6 ± 0.4% *versus* 5.4 ± 0.3% *versus* 5.3 ± 0.2%, respectively, p = 0.006), fasting insulin (13.2 ± 6.6 µIU/mL *versus* 11.1 ± 5.8 µIU/mL *versus* 9.5 ± 4.9 µIU/mL, respectively, p = 0.035), HOMA-IR (3.1 ± 1.7 *versus* 2.5 ± 1.3 *versus* 2.1 ± 1.2, respectively, p = 0.035), and TyG index (4.6 ± 0.2 *versus* 4.5 ± 0.2 *versus* 4.5 ± 0.3, respectively, p = 0.010). The percentages of cases of prediabetes did not differ significantly between groups. Figure 2 presents the mean levels and 95% confidence intervals of fasting and 2-hour plasma glucose levels, fasting insulin, HbA1c, HOMA-IR, and TyG index at 2-4 months after delivery according to NC measured during pregnancy.

Neck circumference tertiles: first tertile (neck circumerence ≤ 33.3 cm), second tertile (neck circumference 33.4-36.3 cm), and third tertile (neck circumference ≥ 36.4 cm). Abbreviations: HbA1c, glycated hemoglobin; HOMA-IR, homeostatic model of insulin resistance; TyG index, triglyceride-glucose index. HOMA-IR = (fasting insulin in µIU/mL x fasting glucose in mmol/L) / 22.5. TyG index = log (fasting triglycerides in mg/dL X fasting glucose in mg/dL) / 2.

In crude linear regression analysis, NC during pregnancy had a significant association with levels of fasting plasma glucose, 2-hour plasma glucose, HbA1c, log HOMA-IR, and TyG index. The association remained after adjustment for age, family history of diabetes, and number of pregnancies (Model 1). When pregestational BMI and weight gain during pregnancy were introduced into the model (Model 2), NC remained independently associated with fasting plasma glucose and HbA1c level. When the models were adjusted for GDM status (Model 3), the association remained with HbA1c and log HOMA-IR but with borderline significance (Table 2).

**Table 2:** Association between neck circumference measured during pregnancy and glucose levels and markers of insulin resistance in the postpartum among women with obesity/overweight with and without GDM

	Fasting glucose (mg/dL)		2-hour glucose (mg/dL)		HbA1c (%)		Fasting insulin (µUI/mL)		Log HOMA-IR		TyG index	
	β (95% CI)	p	β (95% CI)	p	β (95% CI)	p	β (95% CI)	p	β (95% CI)	p	β (95% CI)	p
Crude	1.22 (0.30-2.13)	0.010	3.09 (0.27-5.91)	0.032	0.05 (0.02-0.08)	0.001	0.64 (0.15-1.14)	0.011	0.07 (0.02-0.11)	0.006	0.03 (0.008-0.05)	0.008
Model 1	1.33 (0.43-2.24)	0.004	3.16 (0.47-5.84)	0.022	0.05 (0.02-0.08)	0.002	0.71 (0.20-1.22)	0.006	0.07 (0.03-0.12)	0.003	0.03 (0.009-0.05)	0.006
Model 2	1.42 (0.29-2.55)	0.015	2.48 (-0.79-5.75)	0.136	0.52 (0.1-0.09)	0.006	0.50 (-0.11-1.17)	0.107	0.05 (-0.00-0.11)	0.072	0.02 (-0.006-0.04)	0.138
Model 3	0.90 (-0.26 – 2.07)	0.128	1.08 (-2.31-4.47)	0.529	0.04 (-0.00-0.07)	0.060	0.55 (-1.01-1.21)	0.096	0.05 (-0.01-0.12)	0.085	0.01 (-0.011-0.04)	0.259

Linear regression analysis. Model 1, adjusted for age, family history of diabetes, and number of pregnancies. Model 2, Model 1 plus pregestational body mass index and weight gain during pregnancy. Model 3, Model 2 plus gestational diabetes.

## DISCUSSION

The present study evaluated the association of NC measured during pregnancy and glucose metabolism in the postpartum period. The results showed that NC during pregnancy was directly associated with biomarkers of glucose metabolism and insulin resistance (levels of fasting plasma glucose, 2-hour plasma glucose, HbA1c, fasting insulin, HOMA-IR, and TyG index), which could represent early markers of T2DM risk. The early identification of women at a higher risk of developing T2DM could help improve strategies to prevent this complication.

Excess weight and previous diagnosis of GDM are known risk factors for the development of T2DM (1). Women with a history of GDM have a risk of T2DM 10 times higher than those without changes in plasma glucose levels throughout pregnancy (14). Women with GDM and excess weight are at higher risk of progressing to T2DM over time (5). However, there is a gap in the knowledge of predictive factors identifying women with subclinical alterations of glucose metabolism. Closing this knowledge gap could help guide preventive strategies to avoid or delay the occurrence of T2DM. To date, the use of BMI and waist circumference measurements is recommended to estimate the metabolic risk according to guidelines from scientific societies, with well-defined cutoff points described (13-16). The NC has gained attention from researchers and health care professionals. This marker is particularly interesting in pregnant women during gestation and postpartum, as it is easy to measure and economically feasible in different sociocultural scenarios.

In the present study, we found that NC measured during pregnancy was associated with anthropometric and metabolic parameters in pregnancy. Women in the highest NC tertile (*i.e.*, ≥ 36.4 cm) during pregnancy had greater pregestational and postgestational BMI, higher systolic and diastolic blood pressure levels during pregnancy and postpartum, and greater levels of triglycerides during postpartum. Our results are in line with those of previous studies that observed a positive association between NC and risk factors for metabolic syndrome in adults (*i.e.*, such as excess weight, high blood pressure, insulin resistance, and elevated triglycerides) at different cutoff measurements of NC (5,17-20). Our findings in the present study confirm that NC can be a good marker of metabolic syndrome also in pregnant women.

Regarding the predictive value of NC on diabetes risk, the results of our study showed that NC measured during pregnancy was directly associated with markers of glucose metabolism and insulin resistance. Additionally, NC during pregnancy was significantly associated with levels of fasting plasma glucose, 2-hour plasma glucose, HbA1c, log HOMA-IR, and TyG index. The association remained after adjustment for age, family history of diabetes, and number of pregnancies. When adjusted for other anthropometric parameters related to T2DM (*i.e.*, pregestational BMI and weight gain during pregnancy), NC remained independently and directly associated with levels of fasting plasma glucose and HbA1c in the postpartum. However, the association lost significance after adjustment for the presence of GDM. Considering the natural history of diabetes (GDM or T2DM), adiposity precedes GDM, which in turn predicts changes in glucose metabolism after delivery. Thus, GDM could be a mediator in the association between adiposity (measured herein as NC during pregnancy) and changes in glucose metabolism in the postpartum period. Thus, the loss of significance after adjustment for GDM corroborates the hypothesis that GDM mediates the association between NC and glucose metabolism after delivery. These results call attention to NC – a feasible anthropometric measurement in pregnancy – as a predictive marker for glucose metabolism in the postpartum and, possibly, T2DM risk in the long term.

Evidence on the association between NC and diabetes risk in the general population is scarce in the scientific literature (21-23), and we found no studies that have enrolled pregnant women at a higher risk of T2DM. In contrast, studies assessing NC and risk of diabetes in nonpregnant women have included diverse ethnic populations. The NC was first associated with T2DM in 1989, when Freedman & Rimm reported the occurrence of the association in a study of 43,595 women, which occurred independently of the degree of excess weight (21). A recent prospective cohort evaluating the association between NC and development of diabetes followed a Korean population over 10 years and reported on the probability of developing diabetes according to NC. A Chinese prospective cohort also showed that NC was an independent predictor of T2DM in adults (24). Considering the results of the present study, we hypothesize that NC measured during pregnancy could help improve the prediction of T2DM among women at high risk.

We did not analyze maternal and fetal outcomes according to NC tertiles in the present study. We hypothesized that these outcomes would have other relevant risk factors, such as glucose control and prenatal care, which were very similar for all participants. Notably, all women included in the study underwent frequent evaluations by a multidisciplinary professional group including physicians, nutritionists, obstetricians, and psychologists.

We must point out some limitations of the present study. The study included a small sample size and a short follow-up time in the postpartum period (*i.e.*, 2-6 months), which may not have been long enough to observe the development of T2DM. Additionally, only a few cases of prediabetes were included, although the number of such cases was not different between tertiles (i.e., 7 in the first tertile, 7 in the second tertile, and 9 in the third tertile). Regarding the participants who were lost to follow-up, we compared them with those who were followed up throughout the study period and found no significant differences between groups regarding characteristics during pregnancy (Supplementary Table). Our study has several strengths, including the evaluation spanning the entire pregnancy and in the postpartum period, which ensured reliable data collected by the researchers, precluding memory or information bias. Another relevant point was that the groups with and without GDM had similar pregestational BMI and all the groups included participants who were overweight or obese, allowing the adjustments for this important risk factor associated with NC, glucose metabolism, and insulin resistance biomarkers.

In conclusion, the present study showed that NC measured during pregnancy was directly associated with markers of insulin resistance and glucose metabolism in the postpartum period among women at high risk of progression to T2DM. In some of them, the risk remained even after adjustments for BMI. The results of our study reinforce the evidence that NC could be a feasible tool for the early identification of women at higher risk of developing T2DM, contributing to improving prevention strategies for this population at risk. Evaluation in studies with a longer follow-up period will help define the NC cutoff values in pregnancy in predicting T2DM in the long term.

## Appendix

**Supplementary Table.** Comparison of relevant characteristics between women who attended the postpartum evaluation versus those who were lost to follow-up

	Women who attended the postpartum evaluation (n=100)	Women who were lost to follow-up (n=43)	P values
Age (years)	30.7 ± 6.6	30.2 ± 5.7	0.661
Family history of DM	34 (34.3)	15 (35.7)	0.876
Physical activity	42 (42.0)	19 (45.2)	0.722
Pregestational BMI (kg/m^2^)	29.7 (3.9)	30.0 (3.7)	0.603
Gestational diabetes	50 (50.0)	16 (38.1)	0.194
First pregnancy	30 (30.0)	11 (26.2)	0.155
Systolic BP (mmHg)	113.7 ± 11.7	111.2 ± 10.4	0.343
Diastolic BP (mmHg)	71.0 ± 10.2	68.8 ± 10.1	0.350

The values are shown as mean ± standard deviation or n (%). The variables were analyzed using Student's t test or chi-square test. P values < 0.05 were considered significant. Abbreviations: BMI, body mass index; BP, blood pressure. Physical activity levels were deemed to be of moderate intensity when lasting ≥ 75 minutes/week and of high intensity when lasting ≥ 150 minutes/week.
